# Desloratadine for the Relief of Nasal and Non-nasal Allergy Symptoms: An Observational Study

**DOI:** 10.1111/j.1753-5174.2009.00018.x

**Published:** 2009-06

**Authors:** Werner Aberer

**Affiliations:** Department of Environmental Dermatology, Medical University of GrazGraz, Austria

**Keywords:** Allergy, Allergic Rhinitis, Chronic Urticaria, Desloratadine

## Abstract

**Introduction:**

The rates of allergic rhinitis, allergic asthma, and atopic eczema range from 6% to 16% globally. Second-generation antihistamines have been shown to be safe and effective for the treatment of symptoms of allergic disease. This study investigated the efficacy and safety of desloratadine, a nonsedating second-generation antihistamine, in the treatment of common allergy symptoms.

**Methods:**

In this open-label, uncontrolled, non-randomized, observational study, subjects (N = 973) with allergy symptoms were given desloratadine 5 mg daily for 3 weeks. Nasal, ocular, and dermal symptom severity was rated as asymptomatic, mild, moderate, or severe; changes in the percentage of subjects in each severity category were assessed. Overall efficacy and tolerability of desloratadine treatment were evaluated separately by physicians and subjects.

**Results:**

Allergic rhinitis was the most frequent diagnosis, occurring in 59.0% of subjects. Approximately 40% of subjects had received previous treatment with other antihistamines, systemic/topical glucocorticosteroids, or beta-sympathicomimetics. Slightly more than half of subjects received concomitant medication during the study; 263 (53.0%) of those used intranasal steroids. A significant reduction in severity scores was observed in all symptom subgroups (*P* < 0.001). Desloratadine efficacy was judged to be excellent or good by 90.2% of physicians and 88.6% of subjects; 82.5% of investigators and 80.9% of subjects considered it more effective than previous therapy. The tolerability of desloratadine was rated excellent or good by 97.0% of both groups. Thirty-one subjects (3.2%) experienced adverse events.

**Conclusions:**

In an open-label, uncontrolled, non-randomized, observational study allergy symptoms improved significantly in subjects treated with desloratadine.

## Introduction

The prevalence of allergic disease has increased dramatically worldwide [1]. A survey conducted in 2007 by the World Allergy Organization revealed that the prevalence of allergic rhinitis was greater than 16% in most of the 37 countries reporting and reached as high as 40% in the Ukraine. Rates of allergic asthma ranged from 6% to 15% in 35 countries surveyed, which was also the range of prevalence rates of atopic eczema reported by 30 countries [2].

Allergy symptoms—nasal congestion; nasal itching; rhinorrhea; sneezing; burning, watery eyes; itching skin and wheals—can be debilitating and often impair sleep, work/school productivity, learning, and concentration, significantly reducing quality of life [3–7]. The allergic inflammatory response consists of a complex network of cellular events in which histamine plays a crucial role. Many of the inflammatory and immunomodulatory effects of histamine are mediated through the H_1_-receptor [8,9]. Therefore, H_1_-receptor antagonists are often recommended first-line treatments for conditions involving activation of allergic inflammation [10–13].

Second-generation oral antihistamines, such as desloratadine, fexofenadine, and levocetirizine, rapidly reduce allergic rhinitis–related nasal symptoms (rhinorrhea, sneezing/itching) and ocular symptoms (tearing, itching, redness) as well as dermatologic symptoms of chronic urticaria (wheals, itching) with little or no somnolence [12]. These agents may also inhibit the actions of other mast cell and basophil mediators that cause nasal obstruction and inflammation of nasal mucosa [14].

Desloratadine is a potent, nonsedating H_1_-receptor antagonist with antiallergic and anti-inflammatory properties. *In vitro* studies have shown that desloratadine inhibits chemical mediators involved in both the early- and late-phase allergic responses [12]. Desloratadine has also been found to improve nasal airflow in patients with allergic rhinitis [15]. The proven ability of desloratadine to prevent the release of cytokines, chemokines, and cellular adhesion molecules associated with the late-phase response may contribute to its decongestant properties [12,16–19].

The objectives of this observational study were to evaluate the efficacy of desloratadine in improving nasal, ocular, and dermal allergy symptoms; to assess the drug's adverse event profile; and to determine investigator and subject satisfaction with desloratadine treatment in a real-life environment.

## Methods

This multicenter, observational study was conducted by 85 dermatology specialists and in three hospital allergy units in Austria in subjects aged 12 years or older who were currently experiencing allergy symptoms related to allergic rhinitis (with or without comorbidities such as conjunctivitis, bronchial hyperreactivity, or asthma); sinusitis, laryngitis, or polyposis; chronic idiopathic urticaria; skin or food allergies; or bronchial hyperreactivity or asthma. Written informed consent was obtained from all subjects. According to Austrian law on medicinal products, no official institutional review by the Medical University of Graz was required because, in this study, desloratadine was prescribed according to the indications (allergic rhinitis and chronic idiopathic urticaria) in the label approved at the time. Neither the investigators nor the subjects were paid for their participation in this study. The study drug was supplied free of charge.

Study candidates were excluded if they were pregnant or breastfeeding or were receiving current or continuing treatment with other systemic antihistamines.

The participants received desloratadine 5 mg once daily for 3 weeks. Subjects could also be prescribed concomitant medications, e.g., corticosteroids for nasal congestion, if an investigator felt it was warranted.

Allergy symptoms were assessed at the beginning and end of treatment with desloratadine. Subjects completed daily diaries, categorizing their symptoms into 3 subgroups: nasal (congestion, rhinorrhea, sneezing/itching), ocular (tearing, burning/itching, redness), and dermal (itching, wheals, dryness) and scoring them according to 4 levels of severity: 0 = asymptomatic, 1 = mild, 2 = moderate, or 3 = severe. Subjects were instructed on diary use, and the importance of completing them was stressed. Change between baseline and study endpoint in percentage of subjects in each category of symptom severity were analyzed using the McNemar test and Wilcoxon signed-rank test. Percentages were derived without accounting for missing values. In addition, the overall efficacy of desloratadine therapy was assessed separately by investigators and subjects at study end using 4 descriptors—excellent, good, moderate, or inadequate. Further, investigators who had previously administered allergy treatment to the study subjects, and the subjects who had received such treatment, judged whether desloratadine “was better than prior therapy.”

Tolerability of desloratadine therapy was assessed separately by investigators and subjects at study end using the same 4 descriptors as used for efficacy analysis. Subjects were asked to report all potential adverse effects, including dry mouth, somnolence, headache, or gastrointestinal upset. In addition, subjects described the onset of any new illnesses or exacerbations of existing illnesses after beginning desloratadine treatment.

## Results

### Subjects

A total of 1,015 subjects were enrolled. Data were available for 973 subjects who received desloratadine, some of whom were taking the drug to treat more than one type of allergy. Forty-two subjects did not complete the study; their data were not included in the analysis. No subject discontinued due to lack of response.

The mean age of the study population was 37.5 years, and 58% were women. Allergic rhinitis was identified as the sole type of allergy in 59.2% (N = 574) of subjects and in conjunction with bronchial asthma in another 10.3% (N = 100). Nearly 40% of subjects had received previous allergy treatment with sedating and other nonsedating antihistamines, glucocorticosteroids, or beta-sympathicomimetics (Table 1) for a mean duration of 27.2 days (range 2 to 202 days). A total of 496 subjects (51.0%) received concomitant medications during the study; 263 (53.0%) of those used intranasal steroids. The proportion of comedicated subjects receiving any other class of medication, including topical antihistamines, was <10%.

**Table 1:** Concomitant medications

Medication (multiple responses possible)	Subjects, N (% of total population)
Antiallergic agent (topical)	27 (2.8)
Antibiotics	3 (0.3)
Antidepressant	4 (0.4)
Antihistamine (topical)	38 (3.9)
Beta-sympathicomimetic	39 (4.0)
Glucocorticoid	
Dermal cream/lotion	18 (1.9)
Bronchial	27 (2.8)
Combined	10 (1.0)
Intranasal spray	263 (27.0)
Systemic	9 (0.9)
Hyposensitization	11 (1.1)
Leukotriene inhibitor	9 (0.9)
Local vasoconstrictor	8 (0.8)
Other	80 (8.2)

### Efficacy

The data showed that nasal, ocular, and dermal symptoms all improved significantly following desloratadine treatment (*P* < 0.001).

#### Nasal Symptoms

**Figure 1:**
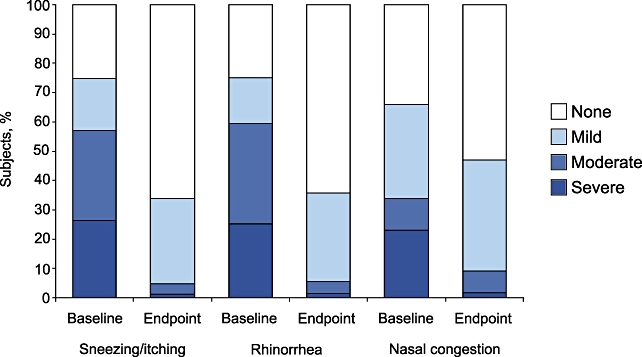
Baseline-to-endpoint changes in severity of nasal symptoms of sneezing/itching (A), rhinorrhea (B), and congestion (C) after 3 weeks of desloratadine treatment. Severity was rated as asymptomatic, mild, moderate, or severe.

The percentage of subjects with no or only mild nasal symptoms increased from 42.9% at baseline to 95.2% after desloratadine treatment for sneezing/itching; from 40.5% to 94.4% for rhinorrhea; and from 33.7% to 90.7% for nasal congestion (Figure 1). The proportion with moderate or severe nasal symptoms decreased from 57.2% at baseline to 4.8% at endpoint in subjects with sneezing/itching; from 59.5% to 5.6% in those with rhinorrhea; and from 66.2% to 9.2% in those with nasal congestion.

#### Ocular Symptoms

**Figure 2:**
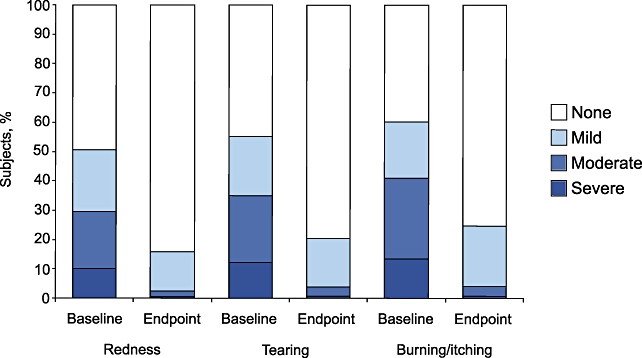
Baseline-to-endpoint changes in severity of ocular redness (A), tearing (B), and burning/itching (C) after 3 weeks of desloratadine treatment. Severity was rated as asymptomatic, mild, moderate, or severe.

From baseline to endpoint, the proportion of subjects with no or only mild ocular symptoms increased from 70.4% to 97.7% for redness; from 65.0% to 96.2% for tearing; and from 59.0% to 95.9% for burning/itching (Figure 2). The proportion of subjects with moderate-to-severe symptoms decreased from 29.6% to 2.3% for redness, from 35.0% to 3.8% for tearing, and from 41.0% to 4.2% for burning/itching.

#### Dermal Symptoms

**Figure 3:**
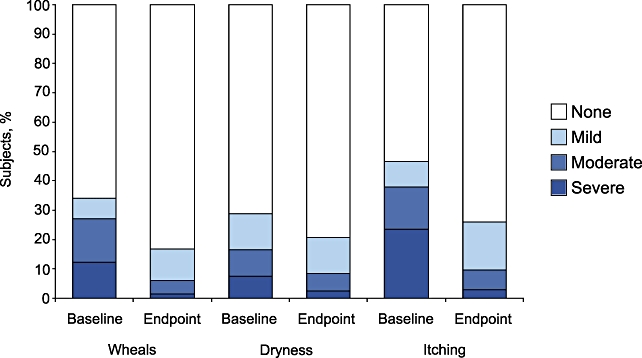
Baseline-to-endpoint changes in severity of wheals (A), dryness (B), and itching (C) after 3 weeks of desloratadine treatment. Severity was rated as asymptomatic, mild, moderate, or severe.

The proportion of subjects with no or mild dermal symptoms increased from 72.8% at baseline to 94.0% after desloratadine therapy for wheals; from 83.6% to 91.6% for dryness, and from 62.2% to 90.5% for itching (Figure 3). The percentages of subjects experiencing moderate and severe dermal symptoms decreased from 27.2% to 6.0% for wheals, from 16.5% to 8.4% for dryness, and from 37.9% to 9.5%% for itching.

### Investigator/Subject Evaluations

**Figure 4:**
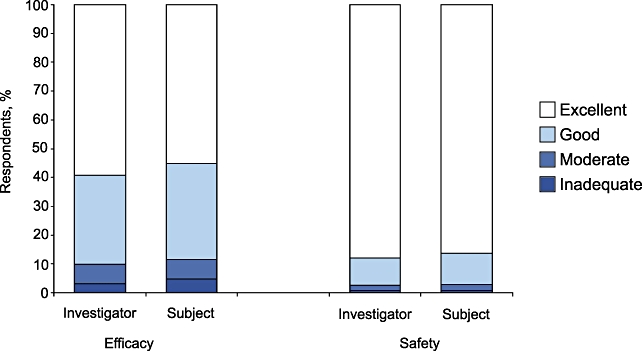
Desloratadine efficacy (A) and tolerability (B) as rated by physicians and subjects at endpoint. Efficacy and tolerability were rated as excellent, good, moderate, or inadequate.

Desloratadine efficacy was determined to be excellent or good by 90.2% of physicians and 88.5% of subjects at the end of therapy (Figure 4). Moreover, approximately 97% of participants in both groups rated the tolerability of desloratadine with these top 2 descriptors. Investigators (82.5%) and subjects (80.9%) described treatment with desloratadine as being better than previous allergy therapy. Of all subjects remaining on any medication at endpoint, 88.0% elected to continue treatment with desloratadine.

### Safety

Adverse events were reported by 3.2% of subjects (N = 31). Fatigue was the most common adverse event (0.8%), followed by dry mouth, gastritis, and headache (0.3% each). No subject discontinued due to adverse effects.

## Discussion

The findings of improved allergy symptoms confirm the results of previous studies indicating that desloratadine is an effective and well-tolerated treatment for the common signs and symptoms of seasonal, perennial, and intermittent allergic rhinitis, including nasal congestion [20–25], and chronic urticaria [26–28]. Desloratadine treatment significantly reduced total nasal and non-nasal symptom scores from baseline and decreased pruritus scores and number and size of wheals associated with moderate-to-severe chronic idiopathic urticaria.

## Limitations

In this study, we used a single agent in an open-label, non–placebo-controlled design. In addition, 51.0% of subjects were receiving concomitant medications, including 263 (27.0%) subjects who were using intranasal steroids. However, this design in a patient population normally seen in clinical practice more closely approximates a physician's everyday experience. Moreover, we cannot infer that the improvements observed would have lasted beyond 3 weeks of the study. It is also possible that, over the course of the study to endpoint, subjects’ symptoms may have abated independent of desloratadine treatment.

## Conclusion

In this open-label, uncontrolled, observational study, treatment with desloratadine resulted in a significant reduction in severity scores in all allergy symptom subgroups. Both investigators and subjects rated desloratadine efficacy as excellent or good.
